# TESC Promotes TGF-α/EGFR-FOXM1-Mediated Tumor Progression in Cholangiocarcinoma

**DOI:** 10.3390/cancers12051105

**Published:** 2020-04-29

**Authors:** Cheng-Han Hsieh, Cheng-Ying Chu, Sey-En Lin, Yu-Chen S.H. Yang, Hung-Shu Chang, Yun Yen

**Affiliations:** 1 TMU Research Center of Cancer Translational Medicine, Taipei Medical University, Taipei 110, Taiwan; 2 CRISPR Gene Targeting Core Lab, Taipei Medical University, Taipei 110, Taiwan; 3 Department of Pathology, Wan Fang Hospital, Taipei Medical University, Taipei 110, Taiwan; 4 Joint Biobank, Office of Human Research, Taipei Medical University, Taipei 110, Taiwan; 5 Department of Physiology, School of Medicine, College of Medicine, Taipei Medical University, Taipei 110, Taiwan; 6 Ph.D. Program for Translational Medicine, College of Medical Science and Technology, Taipei Medical University, Taipei 110, Taiwan; 7 Ph.D. Program for Cancer Molecular Biology and Drug Discovery, College of Medical Science and Technology, Taipei Medical University, Taipei 110, Taiwan; 8 Graduate Institute of Cancer Biology and Drug Discovery, Taipei Medical University, Taipei 110, Taiwan; 9 Cancer Center, Taipei Municipal Wanfang Hospital, Taipei 110, Taiwan

**Keywords:** cholangiocarcinoma, oncogenic pathway, EGFR signaling, TESC, FOXM1

## Abstract

Cholangiocarcinoma is a relatively uncommon but highly lethal malignancy. Improving outcomes in patients depends on earlier diagnosis and appropriate treatment; however, no satisfactory diagnostic biomarkers or targeted therapies are currently available. To address this shortcoming, we analyzed the transcriptomic datasets of cholangiocarcinoma from The Cancer Genome Atlas (TCGA) and Gene Expression Omnibus (GEO) databases and found that TESC is highly expressed in cholangiocarcinoma. Elevated cellular levels of *TESC* are correlated with larger tumor size and predict a poor survival outcome for patients. Knockdown of TESC via RNA interference suppresses tumor growth. RNA-sequencing analysis showed that silencing of TESC decreases the level of FOXM1, leading to cell cycle arrest. Correlation analysis revealed that the cellular level of *TESC* is correlated with that of *FOXM1* in cholangiocarcinoma patients. We further observed that upon TGF-α induction, TESC is upregulated through the EGFR-STAT3 pathway and mediates TGF-α-induced tumor cell proliferation. In vivo experiments revealed that knockdown of TESC significantly attenuates tumor cell growth. Therefore, our data provide novel insight into TESC-mediated oncogenesis and reveal that TESC is a potential biomarker or serves as a therapeutic target for cholangiocarcinoma.

## 1. Introduction

Cholangiocarcinoma, which is a rare malignant tumor, arises from biliary epithelial cells in the biliary tree. Radical surgical resection is an acceptable form of curative treatment. However, there are no noticeable symptoms in the early stage of cholangiocarcinoma [1]; thus, patients are usually diagnosed in the advanced or metastatic stages of the disease, making it difficult to perform surgical resection [2,3,4]. In the past decade, the incidence of cholangiocarcinoma has rapidly increased globally [5], necessitating the development of strategies for early diagnosis and effective treatment.

TESC, also known as tescalcin or calcineurin B homologous protein 3 (CHP3), was originally discovered during an analysis of embryonic mouse testis and contains an EF-hand motif that is characteristic of a large family of Ca^2+^-binding proteins, the members of which play critical roles in several cellular processes [6]. TESC interacts directly with the Na^+^/H^+^ exchanger, thereby modulating cytoplasmic pH [7,8]. Additionally, TESC plays a vital role in megakaryocytic differentiation and maturation through the regulating of the gene expression of E26 transformation-specific transcription factors [9]. Several recent reports demonstrated that overproduction of cellular TESC is linked to tumor progression of several types of cancer, including radiation-induced papillary thyroid carcinoma, acute myeloid leukemia, renal cell carcinoma, and human colorectal cancer [10,11,12,13,14]. However, the regulatory signaling and mechanisms underlying TESC-mediated oncogenesis are not well understood. 

Aberrant regulation of the cell cycle has been implicated in cancer progression [15,16]. FOXM1, a member of the Forkhead transcription factor family, regulates a number of genes, such as *CCNB1*, *PLK1, CDC25B, CDC25C, BIRC5*, and *AURKB*, which are critical for cell cycle progression—particularly during the G2/M transition [17,18,19]. The expression of FOXM1 is detected during the G1 phase, and then its cellular concentration gradually increases, reaching a maximum during G2/M [20]. Abnormal expression of FOXM1 is detected in a variety of human cancers, including colorectal, lung, prostate, liver, and breast carcinomas, associated with cancer progression [21]. Elevated cellular levels of FOXM1 in tumors have been strongly correlated with poor prognosis for cancer patients.

In this study, we addressed the role of TESC in cholangiocarcinoma tumorigenesis, finding that the TESC level is markedly elevated in cholangiocarcinoma and involved in FOXM1-mediated tumor development. TESC was induced by TGF-α/EGFR signaling in cholangiocarcinoma. We further examined the biological and clinical significance of the TESC–FOXM1 axis in cholangiocarcinoma.

## 2. Results

### 2.1. Identification of Genes Upregulated and Associated with Overall Survival (OS) in Cholangiocarcinoma

**Figure 1:**
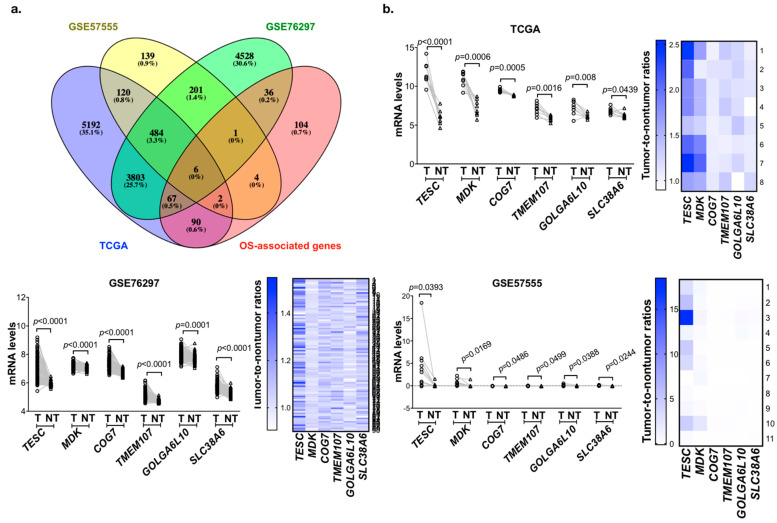
Identification of candidate genes that are upregulated in cholangiocarcinoma and associated with poor survival. (**a**) Venn diagram of overall survival (OS)-associated gene analysis in The Cancer Genome Atlas (TCGA) and differentially expressed genes in the TCGA and Gene Expression Omnibus (GEO) databases. (**b**) Line plots of differential candidate genes transcript expression in paired tumor (T) and matched adjacent non-tumor (NT) tissues from the TCGA (upper), GSE76297 (bottom left,) and GSE57555 (bottom right). Heat map representing tumor-to-non-tumor ratios of candidate genes from TCGA (upper), GSE76297 (bottom left), and GSE57555 (bottom right) datasets. *p*-values indicate significant differences as determined by the Wilcoxon matched pairs test.

**Figure 2:**
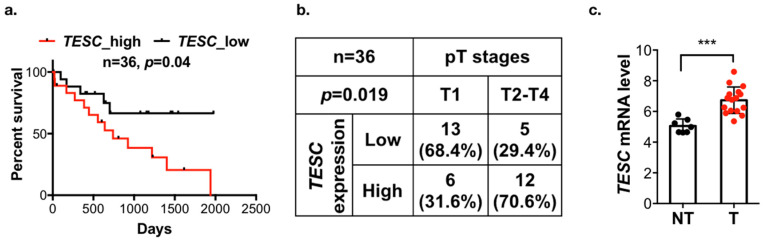
*TESC* is associated with poor outcomes in patients with cholangiocarcinoma. (**a**) Kaplan–Meier analysis of *TESC* expression with overall survival in 36 cholangiocarcinoma patients from the TCGA database. (**b**) Chi-square analysis of the association between *TESC* expression and pT stages in cholangiocarcinoma from the TCGA database. (**c**) Scatter plots of *TESC* expression in tumor (T) versus adjacent non-tumor tissues (NT) from the GSE32879 database, which contains 16 intrahepatic cholangiocarcinoma (ICC) samples and 7 NT samples. *** *p* < 0.001.

To identify the potential genes involved in tumorigenesis and predict poor survival in cholangiocarcinoma, we analyzed the gene expression profiling datasets of cholangiocarcinoma from the The Cancer Genome Atlas (TCGA) and Gene Expression Omnibus (GEO; GSE76297 and GSE57555) databases. These three datasets contain 8, 90, and 11 pairs of intrahepatic cholangiocarcinoma (ICC) tumor tissues and matched adjacent non-tumor tissues. We analyzed the matched tumor–normal pairs and selected the candidate genes that were upregulated in tumor tissues and the threshold was set to *p* < 0.05. After intersecting the results from the different data sources and survival-associated genes based on 36 samples from TCGA, a total of six genes were determined (Figure 1a). Their expression levels are shown in Figure 1b, and the Kaplan–Meier analysis is shown in Figure 2a and Figure S1a. Among these six candidate genes, the expression level of *TESC* was the most significantly different between tumor tissues and matched adjacent non-tumor tissues, and only *TESC* expression was found to be correlated with tumor size (Figure 2b and Figure S1b). According to TCGA database, *TESC* showed the highest expression level in cholangiocarcinoma compared with other types of cancer (Figure S2a). *TESC* expression in different pathological subtypes of cholangiocarcinoma was further examined, and we observed that *TESC* is highly expressed in ICC (Figure S2b). A similar result with high *TESC* expression in ICC was also observed in another cholangiocarcinoma dataset from GSE32879 (Figure 2c). We also examined *CHP1* and *CHP2*, which belong to calcineurin B homologous proteins (CHPs) and share substantial sequencing with TESC, in ICC. The results indicate that *CHP1* and *CHP2* are not highly expressed in ICC versus normal tissues (Figure S3). Therefore, we focused on exploring the role of TESC in ICC.

### 2.2. Participation of TESC in Cholangiocarcinoma

**Figure 3:**
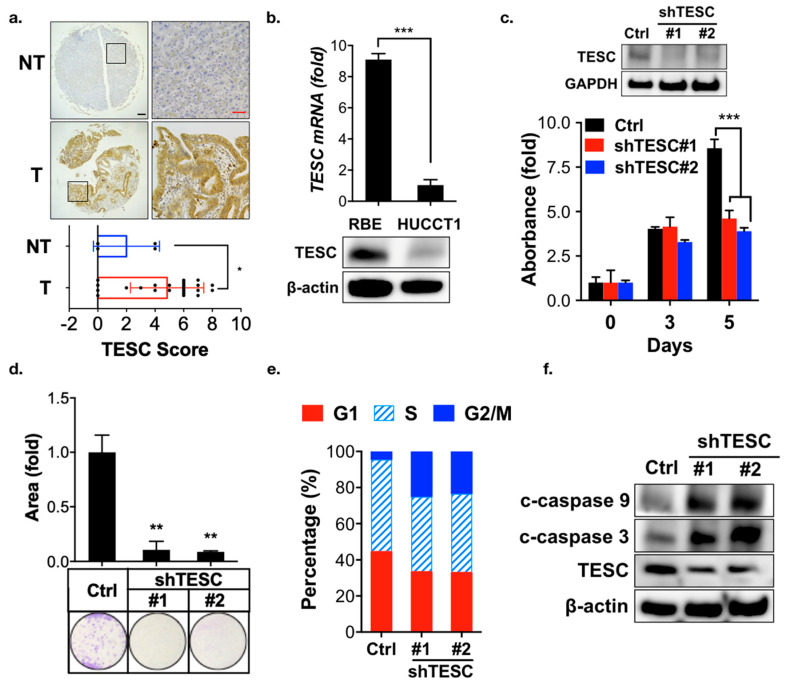
Participation of TESC in ICC oncogenesis. (**a**) Representative images of immunohistochemical staining for TESC expression in tumors (T), with a non-tumor (NT) tissue as a negative staining control (upper). Scatter plot shows differential expression of TESC in tumor (T) and non-tumor (NT) tissues (bottom). Black scale bar, 150 μm; red scale bar, 50 μm. * *p* < 0.05. (**b**) qPCR (upper) and immunoblotting (bottom) analysis of TESC expression in RBE and HUCCT1 cells. Results are representative of three independent experiments and expressed as the mean ± SD; *** *p* < 0.001. (**c**) MTT (3-[4,5-Dimethylthiazol-2-yl]-2,5-diphenyltertrazolium bromide) analysis of proliferation of RBE cells infected with lentiviral vectors encoding shTESC or scrambled control (Ctrl). The level of TESC was assessed through immunoblotting (upper). #1 and #2 indicate distinct shRNAs targeting different regions within TESC. The results are representative of three independent experiments and expressed as the mean ± SD; *** *p* < 0.001. (**d**) Clonogenic assay of RBE cells infected with lentiviral vectors encoding shTESC or scrambled control for 14 days. Top: colonies were stained with crystal violet and quantified. Bottom: representative plates were photographed. ** *p* < 0.01. (**e**) Cell cycle analysis of the loss of the TESC effect on the cell cycle in RBE cells infected with lentiviral vectors encoding shTESC or scrambled control for 5 days. (**f**) Immunoblotting analysis was used to assess the effect of TESC silencing on cleaved (c)-caspase 3 and c-caspase 9 expression in RBE cells infected with lentiviral vectors encoding shTESC or scrambled control (Ctrl).

To investigate the role of TESC in cholangiocarcinoma tumorigenesis, a rat model of caerulein-induced bile-duct lesions was employed [22]. Immunohistochemical analysis revealed that the TESC expression was higher in the caerulein treatment group compared with the non-treatment group (Figure S4). To further confirm the TESC expression pattern in ICC, we performed immunohistochemical staining to detect TESC protein in ICC and non-tumor tissue samples. TESC expression was higher in ICC compared with non-tumor tissue (Figure 3a). These results suggest the possible involvement of TESC in ICC tumorigenesis.

To identify suitable ICC cell lines for functional analysis of TESC, quantitative PCR (qPCR) and immunoblotting analysis were conducted. TESC was highly expressed in RBE cells, whereas HUCCT1 cells contained low levels of TESC (Figure 3b). To determine whether TESC is required for ICC development, TESC was knocked down in RBE and HUCCT1 cells using shRNAs against TESC, and the effect was assessed. MTT (3-[4,5-Dimethylthiazol-2-yl]-2,5-diphenyltertrazolium bromide) and clonogenic assays indicated that TESC silencing significantly attenuated cell proliferation (Figure 3c,d; Figure S5a,b). In contrast, ectopic expression of TESC in HUCCT1 cells enhanced cell proliferation (Figure S5c). Cell cycle analysis revealed that knockdown of TESC results in G2/M cell cycle arrest in RBE and HUCCT1 cells (Figure 3e and Figure S5d). TESC silencing inhibited the ability of migration (Figure S6) and induced apoptosis (Figure 3f). Collectively, these results suggest that TESC participates in ICC tumorigenesis.

### 2.3. TESC Regulates G2/M Phase Through FOXM1

**Figure 4:**
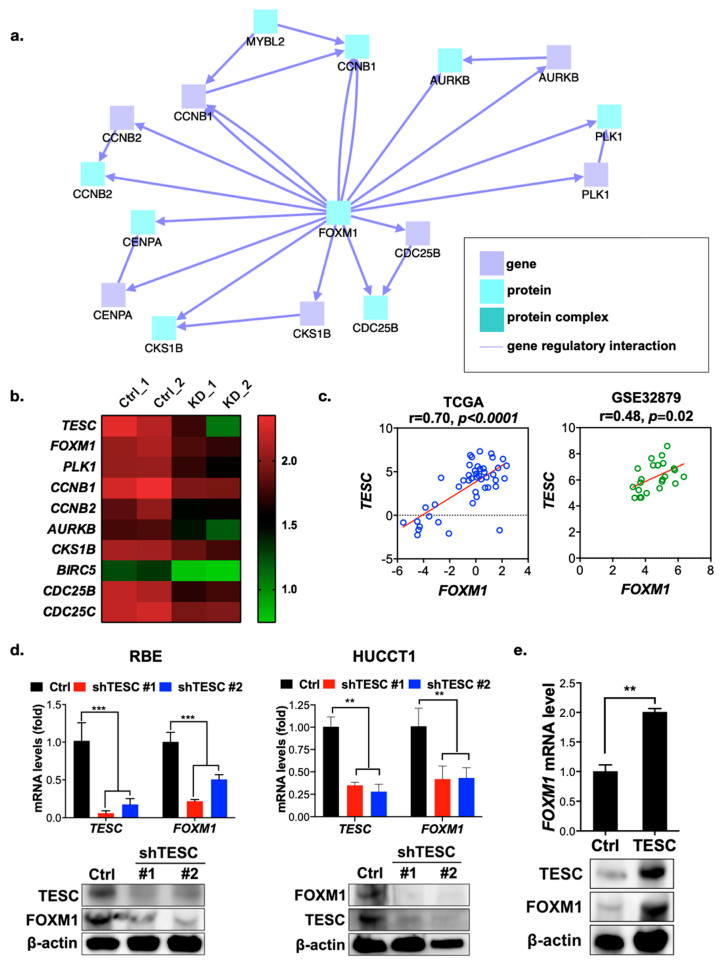
TESC regulates FOXM1 and its downstream genes. (**a**) Network analysis of differentially downregulated genes in TESC-silenced cells. (**b**) Heat map representing expression levels of *FOXM1* and its downstream genes in RBE cells infected with lentiviral vectors encoding shTESC (KD) or lentiviral vectors control (Ctrl). Ctrl_1 and Ctrl_2 indicate different shRNAs that target scrambled and luciferase, respectively. KD_1 and KD_2 indicate distinct shRNAs that target different regions within *TESC*. (**c**) Dot-plot analysis of co-expression between *TESC* and *FOXM1* in cholangiocarcinoma from the TCGA (left) and GSE32879 (right) databases. (**d**) qPCR (upper) and immunoblotting (bottom) analysis of the effect of TESC silencing on FOXM1 expression in RBE (left) and HUCCT1 (right) cells infected with lentiviral vectors encoding shTESC or scrambled control (Ctrl). ** *p* < 0.01, *** *p* < 0.001. (**e**) qPCR (upper) and immunoblotting (bottom) analysis of the effect of TESC overexpression on FOXM1 expression in HUCCT1 cells infected with lentiviral vectors encoding *TESC* cDNA or empty control vector (Ctrl). ** *p* < 0.01.

To understand the molecular mechanism of growth regulation mediated by TESC in ICC, we conducted gene expression profiling in TESC-silenced RBE cells versus control cells by RNA sequencing. We then used the ConsensusPathDB database to complete an induced network module analysis of genes downregulated in TESC-silenced cells. The results reveal a single major gene network in which FOXM1 is the major hub gene (Figure 4a) that connects with a number of genes that regulate the G2/M transition (Figure 4b). We further assessed the possible involvement of FOXM1 in TESC-regulated oncogenesis. Clonogenic assays revealed that knockdown of FOXM1 attenuates the proliferation of RBE cells (Figure S7a). Similar to the results of TESC knockdown, FOXM1 silencing attenuated cell proliferation via G2/M cell cycle arrest (Figure S7b). Gene set enrichment analysis showed that knockdown of TESC led to the downregulation of a number of genes involved in the G2/M transition, and in the FOXM1 pathway particularly (Figure S8a,b). Dot-plot analysis confirmed that the *TESC* level is positively correlated with the level of *FOXM1* and with factors encoded by its downstream genes (Figure 4c and Figure S8c). We confirmed the RNA sequencing results with qPCR and immunoblotting, revealing that the FOXM1 level was lower in TESC-silenced RBE as well as HUCCT1 cells and higher in TESC-overexpressing HUCCT1 cells (Figure 4d,e). These results indicate that TESC regulates the cell cycle during the G2/M transition via FOXM1.

### 2.4. TESC Is Induced by TGF-α/STAT3 Signaling

**Figure 5:**
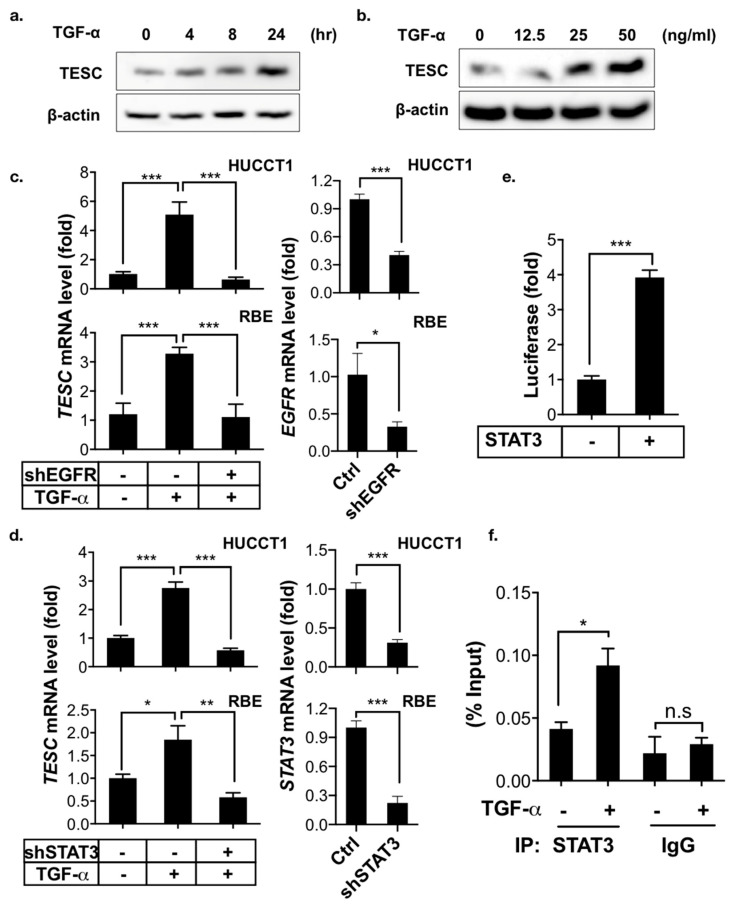
TESC is induced by TGF-α. (**a**) Immunoblotting for TESC in HUCCT1 cells treated with or without TGF-α (50 ng/mL) for the indicated times. β-actin was assessed as an internal control. (**b**) Immunoblotting for TESC in HUCCT1 cells treated with or without TGF-α (50 ng/mL) for the indicated concentrations for 8 h (bottom). β-actin was assessed as an internal control. (**c**) qPCR analysis of *TESC* expression in HUCCT1 (upper) as well as RBE (bottom) cells infected with or without shEGFR in the presence or absence of TGF-α (50 ng/mL) for 4 h. The levels of *EGFR* were determined by qPCR (right). * *p* < 0.05, *** *p* < 0.001. (**d**) qPCR analysis of *TESC* expression in HUCCT1 (upper) as well as RBE (bottom) cells infected with or without shSTAT3 in the presence or absence of TGF-α (50 ng/mL) for 4 h. The levels of *STAT3* were determined by qPCR (right). * *p* < 0.05, ** *p* < 0.01, *** *p* < 0.001. (**e**) Luciferase reporter assay to measure the effect of STAT3 on *TESC* promoter activity in HEK293T cells transfected with the pGL3-*TESC* promoter reporter plus or minus *STAT3* cDNA. *** *p* < 0.001. (**f**) ChIP analysis of STAT3 binding to the *TESC* promoter. HUCCT1 cells treated with or without TGF-α (50 ng/mL) under serum-free conditions for 4 h were subjected to ChIP with an antibody against STAT3, followed by qPCR to assess the binding of STAT3 on the *TESC* promoter. * *p* < 0.05, n.s.: no significance.

Cholangiocarcinoma is a type of cancer arising from the epithelial cells of the bile duct, which delivers bile acid from the liver to the intestinal lumen. Bile acids stimulate the proliferation of cholangiocytes and thereby promote cholangiocarcinoma by transactivating EGFR through a TGF-α-dependent pathway [23]. Signal transducer and activator of transcription 3 (STAT3) is a key mediator of the oncogenic effects caused by EGFR signaling in cholangiocarcinoma [24]. We therefore evaluated the effects of TGF-α/EGFR-STAT3 signaling on TESC expression in ICC. The immunoblotting results demonstrate that TGF-α induced TESC expression in a time- and dose-dependent manner (Figure 5a,b), whereas inhibition of the TGF-α/EGFR-STAT3 signaling pathway by shRNAs targeted against EGFR or STAT3 blocked TGF-α-induced *TESC* expression (Figure 5c,d). Consistently, pharmacologic inhibition of EGFR as well as STAT3 with gefitinib, an EGFR inhibitor, and WP1066, a STAT3 inhibitor, respectively, attenuated the TGF-α-induced upregulation of TESC (Figure S9).

To study the molecular mechanism of TESC regulated by STAT3, the promoter region (1.5 kb upstream) of *TESC* was cloned and used for a promoter reporter assay. The results reveal that STAT3 activated *TESC* transcription (Figure 5e). A possible STAT3-binding site in the −374 to −383 bp region of *TESC* promoter was identified by analyzing *TESC* promoter sequencing. The chromatin immunoprecipitation (ChIP) assay showed that TGF-α enhanced the binding of STAT3 to the predicted *TESC* promoter region (Figure 5f). In summary, these results indicate that TGF-α induces TESC expression via STAT3 binding directly to and activating the *TESC* promoter.

### 2.5. TESC Enhances TGF-α-Induced Cell Proliferation Via FOXM1

**Figure 6:**
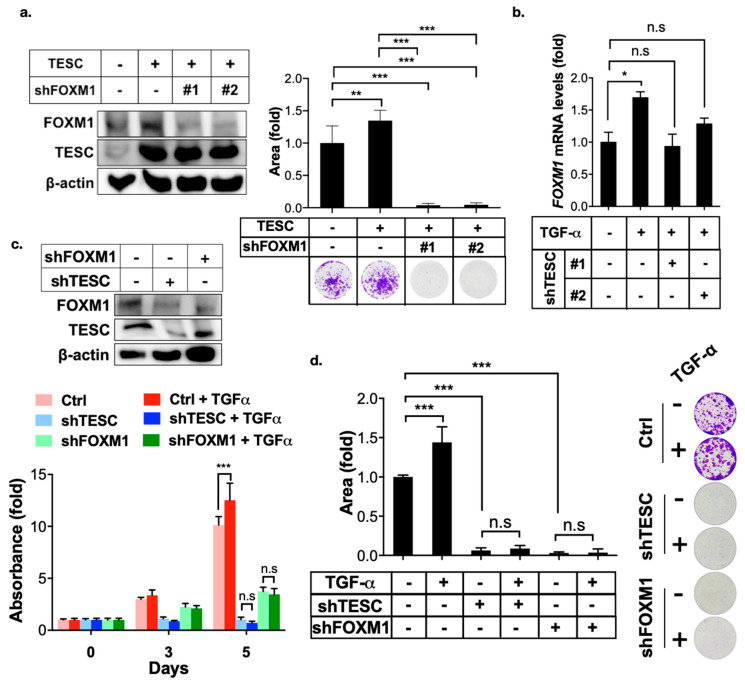
TESC mediates TGF-α-induced FOXM1 expression. (**a**) Clonogenic assays were employed to assess the effect of FOXM1 silenced on TESC-overexpressed HUCCT1 cells. HUCCT1 cells were first infected with lentiviral vectors encoding TESC or vector control for 5 days, followed by infection with lentiviral vectors encoding shTESC or scrambled control for 2 weeks. The levels of TESC and FOXM1 were determined by immunoblotting (left). Colonies were stained with crystal violet and quantified (upper right). Representative plates were photographed (bottom right). ** *p* < 0.01, *** *p* < 0.001. (**b**) qPCR analysis of *FOXM1* expression in HUCCT1 cells infected with or without shTESC in the presence TGF-α (50 ng/mL) for 4 h. * *p* < 0.05, n.s.: no significance. (**c**) MTT assay of proliferation of HUCCT1 cells infected with shTESC as well as shFOXM1 or control vector in the presence or absence of TGF-α (50 ng/mL). The levels of TESC and FOXM1 were determined by immunoblotting (upper). *** *p* < 0.001, n.s.: no significance. (**d**) Clonogenic assays of HUCCT1 cells infected with lentiviral vectors encoding shTESC, shFXOM1, or scrambled control in the presence or absence of TGF-α (50 ng/mL) for 2 weeds. Left: Colonies were stained with crystal violet and quantified. Right: Representative plates. *** *p* < 0.001, n.s.: no significance.

EGFR-STAT3 signaling induces FOXM1 expression [25,26]. Therefore, we examined the effect of TESC in TGF-α/EGFR-mediated FOXM1 expression. The clonogenic assay revealed that TESC promoted cell growth, whereas FOXM1 silencing blocked TESC-mediated cell growth (Figure 6a). Silencing of TESC blocked TGF-α-mediated FOXM1 expression (Figure 6b). The MTT and clonogenic assays indicated that TGF-α treatment promoted cell proliferation, whereas TESC as well as FOXM1 silencing suppressed TGF-α-induced cell proliferation (Figure 6c,d). Our findings support the notion that TESC induces FOXM1 and the TESC–FOXM1 signaling axis involved in TGF-α-mediated cell proliferation. 

### 2.6. TESC Mediates ICC Tumor Growth In Vivo

**Figure 7:**
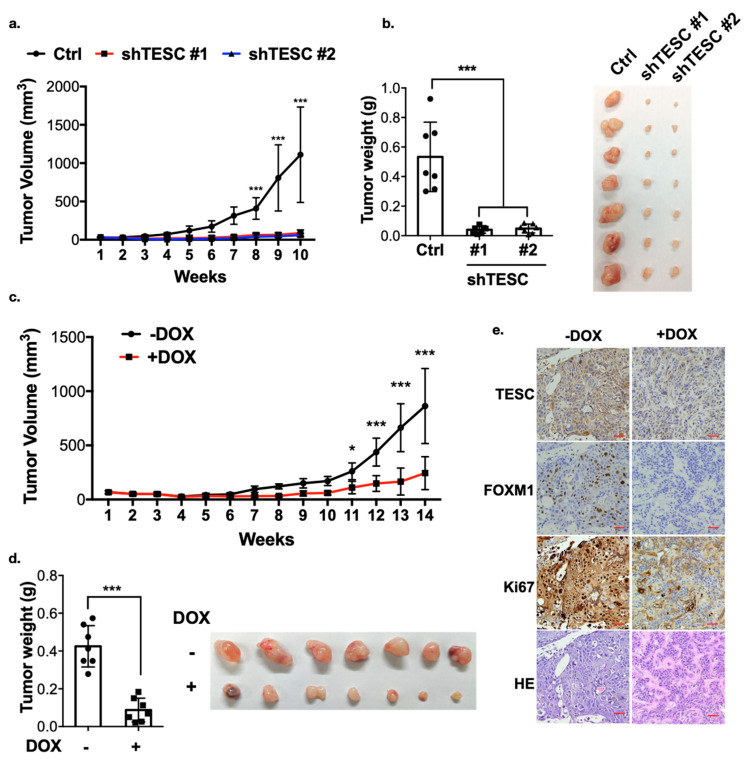
TESC regulates cholangiocarcinoma growth in vivo. (**a**) TESC-silenced HUCCT1 cells were injected subcutaneously into the flank region of nude mice, and tumor volume was monitored. Error bars indicate standard error of the mean (SEM); *n* = 7; *** *p* < 0.001. (**b**) Tumors from (**a**) were weighed (left) and photographed (right) 10 weeks after injection. Error bars indicate SEM; *n* = 7; *** *p* < 0.001. (**c**) HUCCT1-tet_on-shTESC cells were injected subcutaneously into the flank region of nude mice. Two weeks after injection, mice were treated with or without doxycycline (DOX, 1 mg/mL in drinking water). Tumor volumes were monitored over time as indicated. Error bars indicate SEM; *n* = 7; * *p* < 0.05, *** *p* < 0.001. (**d)** Tumors from (**c**) were weighed (left) and photographed (right) 14 weeks after injection. Error bars indicate SEM; *n* = 7; *** *p* < 0.001. (**e**) Excised tumors from mice in (**d**) were further subjected to hematoxylin and eosin (H&E) staining combined with immunohistochemistry analysis. Red scale bar, 50 µM.

We next examined whether TESC is crucial for tumor development. TESC-silenced HUCCT1 cells were injected into nude mice, and tumor volume was monitored over time. The results demonstrate that knockdown of TESC significantly attenuated tumor growth and reduced tumor weight (Figure 7a,b), suggesting that TESC plays an important role in ICC progression. To examine the expression of FOXM1, subcutaneously implanted tumors were excised and analyzed by immunohistochemistry (IHC). We found that the level of FOXM1 was lower in TESC-silenced tumors (Figure S10). The level of Ki67 was also lower in TESC-silenced group, indicating the proliferation ability was suppressed (Figure S10). To further examine the significance of endogenous TESC in tumor formation, we generated inducible TESC-knockdown cells (HUCCT1-pLKO-tet_on-shTESC). Both TESC level and tumor growth markedly decreased after treatment with doxycycline in vitro (Figure S11). HUCCT1-pLKO-tet_on-shTESC cells were also injected into nude mice. Once palpable tumors developed, shTESC was induced in the xenograft tumors through doxycycline treatment. Notably, the knockdown of endogenous TESC in response to doxycycline caused significant tumor shrinkage (Figure 7c,d). The IHC assay showed that the levels of TESC, FOXM1, and Ki67 were downregulated following doxycycline treatment (Figure 7e). Collectively, these results support the hypothesis that TESC regulates ICC development and may therefore represent a therapeutic target.

## 3. Discussion

Cholangiocarcinoma is a cancer with rapidly rising incidence and mortality, but its early diagnosis and treatment are challenging. An available biomarker for the early diagnosis of cholangiocarcinoma is required. In this study, we observed that not only compared with normal biliary tissues but also with other types of cancer, the expression of TESC dramatically increased in cholangiocarcinoma and was associated with poor prognosis of patients with cholangiocarcinoma. These results suggest that TESC may serve as a novel biomarker for cholangiocarcinoma. 

The sequence similarity between TESC and calcineurin B–homologous proteins 1 and 2 (CHP1 and CHP2) is substantial [27,28]. CHP1 is expressed ubiquitously in all tissues [29,30], whereas CHP2 is expressed in the small intestine and several cancers, including hepatic carcinoma, leukemia, breast cancer, and ovarian cancer [31,32,33,34,35]. However, unlike the expression of *TESC*, we found that *CHP1* is dominantly expressed in normal tissues (Figure S3a) and that the expression of *CHP2* does not differ significantly between cholangiocarcinoma and adjacent non-tumor tissues (Figure S3b). These results imply that TESC, and not the other CHP members, plays a specific and vital role in ICC.

Unlike other Ca2^+^-sensor proteins, which mediate short-term physiological responses such as neurotransmitter release or muscle contraction [36,37], TESC serves as a constitutive regulator of gene expression and is involved in physiological processes such as cell differentiation and proliferation [6,38]. Although TESC expression has also been reported in various types of cancer and potentiates cell proliferation and tumorigenicity, the mechanism behind TESC-mediated oncogenesis is unclear. In this study, we observed that TESC enhanced the FOXM1-mediated G2/M phase transition. Knockdown of TESC as well as FOXM1 inhibited tumor cell proliferation and led to G2/M phase arrest. These results suggest a mechanism through which TESC contributes to cholangiocarcinoma development.

TESC expression in tumor cells relies on the methylation status of the H3K9 promoter [39], but the relationship between the tumor microenvironment and the regulatory mechanism of TESC is not well understood. A previous study demonstrated that accumulation of bile acids during cholangiocarcinoma progression leads to the activation of TGF-α/EGFR signaling [23]. EGFR signaling plays a critical role in cholangiocarcinoma development [40,41]. Our present study showed that TGF-α/EGFR signaling induces TESC expression. These results suggest that TESC expression is not only dependent on epigenetic regulation but is also mediated by cytokines. We also found that STAT3, which mediates EGFR signaling, binds directly to the *TESC* promoter and enhances *TESC* transcription. A previous study demonstrated that TESC promoted STAT3 activation, enhancing STAT3-mediated transcription [42]. This implied that a positive feedback loop may exist in TESC regulation. Taken together, these data suggest that the TESC level is tightly regulated by various mechanisms, and aberrant expression of TESC caused by misregulation accelerates tumor progression in cholangiocarcinoma.

In the current study, the inhibition of EGFR signaling by tyrosine kinase inhibitor is considered a potential therapeutic in cholangiocarcinoma; however, the results are not satisfactory [43]. One possible cause is due to the hyper-activation or hyper-expression of downstream molecules. Based on our findings, whether TESC plays a vital role in drug resistance of EGFR tyrosine kinase inhibitor should be further investigated. We plan to examine this issue in a future study.

## 4. Materials and Methods

### 4.1. Reagents

The reagents used in this study are listed in Table S1.

### 4.2. Cell Culture

Two human cholangiocarcinoma cell lines (RBE and HUCCT1) were purchased from RIKEN Bioresource Center (Tsukuba, Ibaraki, Japan) and cultured in RPMI-1640 medium containing 10% fetal bovine serum in a 5% CO_2_ incubator at 37 °C. 

### 4.3. Reverse Transcription-Quantitative PCR 

Total RNA was extracted by using TRIZOL reagent (Invitrogen, Thermo Fisher Scientific, Inc., Waltham, MA, USA) following the manufacturer’s instructions. The cells were incubated with TRIZOL for 5 minutes, then mixed well with BCP (1-Bromo-3-chloropropane, Sigma-Aldrich, St. Louis, MO, USA). After centrifugation, the colorless upper aqueous phase was mixed well with isopropanol (Sigma-Aldrich, St. Louis, MO, USA) to extract total RNA. For reverse transcription-quantitative PCR, 1 µg of total RNA was converted to cDNA by using PrimeScript cDNA synthesis kit (Takara Bio, Shiga, Japan). Reverse transcription-quantitative PCR was performed in triplicate with TOOLS SYBR qPCR Mix Kit (Biotools, Taipei, Taiwan) on Rotor-Gene Q (Qiagen, Hilden, Germany). Relative gene expression was analyzed using the 2^–ΔΔCT^ method. 18S rRNA was used as a reference transcript. Table S2 lists the primer sequences designed to detect specific genes.

### 4.4. MTT Assay

Tumor cells (1 × 10^3^) were seeded in a 96-well plate and cultured for the indicated times. Then, 20 μL of solution containing 5 mg/mL MTT (3-(4,5-dimethylthiazol-2-yl)-2,5-diphenyltetrazolum bromide; Sigma-Aldrich, St. Louis, MO, USA) was added to each well with incubation at 37 °C for 2 h. The supernatant was then discarded, and 100 μL dimethyl sulfoxide (DMSO; Sigma-Aldrich, St. Louis, MO, USA) was added to each well. The optical density was measured at a wavelength of 570 nm. 

### 4.5. Clonogenic Assay

Cells were seeded in six-well plates at 1000 cells/well and incubated for 2 weeks. Colonies were fixed and stained with staining buffer containing 3.7% formaldehyde, 80% methanol, and 0.25% crystal violet (Fisher Scientific, Fairlawn, NJ, USA). The colonies were quantified by measuring the area of colonies using ImageJ software (National Institute of Health, Bethesda, MD, USA). 

### 4.6. Luciferase Reporter Assay

The luciferase reporter assay was conducted using a Dual Luciferase Reporter Assay kit (Promega, Madison, WI, USA) in accordance with the manufacturer’s instructions. pRL-SV40, a renilla luciferase plasmid, was co-transfected into cells and served as an internal control.

### 4.7. Chromatin Immunoprecipitation (ChIP)

ChIP was performed using the Magna ChIP A/G kit (EMD Millipore, Burlington, MA, USA) following manufacturer’s recommendations, and quantified by qPCR. Anti-STAT3 antibody (79D7) used in ChIP experiment was purchased from Cell Signaling Technology (Danvers, MA, USA). Table S3 lists the specific primers used for ChIP-qPCR.

### 4.8. Co-Immunoprecipitation Assay and Immunoblotting

Co-immunoprecipitation assays and immunoblotting were carried out as described [44]. Table S4 lists the antibodies used. The files of immunoblotting with densitometry data can be found in Figure S12.

### 4.9. Plasmid Construction

Human *TESC* cDNA was PCR amplified from the cDNA library and subcloned into HR’-puro describing in previous study [45]. STAT3 was PCR amplified from *STAT3* cDNA and subcloned into pcDNA3. pLKO.1-shEGFR was obtained as previous described [46]. pLKO.1-shTESC, pLKO.1-shFOXM1, and pLKO.1-shSTAT3 were obtained from the National RNAi Core Facility, Academia Sinica (Taipei, Taiwan), and the target sequences for each gene can be found in Table S5. The *TESC* promoter reporters were cloned from genomic DNA and subcloned into pGL3-Basic (Promega, Madison, WI, USA).

### 4.10. Xenograft Tumorigenicity Assay

For tumor implantation, HUCCT1 cells were first infected with lentiviral or doxycycline-inducible lentiviral vectors expressing a short hairpin RNA (shRNA) targeting TESC. One million infected cells were then injected subcutaneously into the flank region of 4-week-old female BALB/c nude mice (*n* = 7 per group). In the experiment for doxycycline-mediated expression of shRNA, mice received 5% sucrose only or 5% sucrose plus 1 mg/mL doxycycline (for control and knockdown cohorts, respectively) 1 week after injection. All water bottles were changed twice a week. All mice were sacrificed by anesthetic overdose at the indicated times. All subcutaneous tumors were measured with calipers every 7 days after injection, and tumor volumes were calculated as width^2^ × (length/2). To assess the effect of caerulein on cholangiocarcinoma tumorigenesis, 6-week-old rats received caerulein (20 μg/kg) by intraperitoneal injection 20 times during the treatment period (*n* = 2 per group). All rats were sacrificed 30 days after treatment. All animals were purchased from BioLASCO (Taipei, Taiwan) and experiments were performed in an accredited facility in accordance with the animal guidelines of the Institutional Animal Care and Use Committee (IACUC) and approved by the Affidavit of Approval of Animal Use Protocol, Taipei Medical University (permit number: LAC-2018-0268).

### 4.11. Immunohistochemistry

Immunohistochemistry was carried out as previously reported [47] using an antibody against TESC (11125-1-AP, 1:50, Proteintech, Rosemont, IL, USA), FOXM1 (D3F2B, 1:50, Cell Signaling Technology, Danvers, MA, USA) and Ki67 (8D5, 1:400, Cell Signaling Technology, Danvers, MA, USA). Sections were dewaxed and subjected to heat-based antigen retrieval with 10 mM sodium citrate buffer (pH 6.0) at 121 °C.

### 4.12. Cell Cycle Analysis 

Cholangiocarcinoma cells were infected with viruses carrying pLKO.1-shTESC, pLKO.1-FOXM1, or pLKO.1-Screamble control. After 5 days’ infection, cells were trypsinized and resuspended at 1 × 10^5^ cells/mL in RPMI-1640 medium. After two ice-cold washes with PBS, cells were fixed in 70% ice-cold ethanol. Before flow cytometry, fixed cells were centrifuged, resuspended in 100 μL PBS containing 0.02 mg/mL RNase H, and stained with 0.05 mg/mL propidium iodide (Sigma-Aldrich, St. Louis, MO, USA). Cell cycle analysis was conducted using a FACSCalibur flow cytometer (BD Biosciences, San Jose, CA, USA) and analyzed using FlowJo software (TreeStar, Stanford, CA, USA).

### 4.13. Wound Healing Assay

Cell were seeded in culture inserts (SPL Life Science, Gyeonggi-do, Korea) at 2 × 10^4^ on 6-well plates. After 24 h of incubation, a cell-free gap was formed by detaching the culture insert. Cell migration toward the gap region was photographed at 0 and 20 h, and quantified by measureing the area of cell migration using Image J software (National Institute of Health, Bethesda, MD, USA). The wound closure was determined as the area migrated after 20 h relative to the area at 0 h.

### 4.14. Patients and Tissue Samples

Cholangiocarcinoma tissue microarrays were purchased from US Biomax (GA802, Rockville, MD, USA). The tissue microarray used in this study contained 25 ICC samples (total amount of ICC samples was 27, but No. 19 and No. 75 were lost) and 4 liver tissue and portal areas (total amount of liver tissue and portal area samples was 5, but No. 76 was lost). The tissue microarray was interpreted and scored according to the intensity and proportion of staining by pathologists. In terms of proportion, 0 points indicated no positive tumor cells, 1 point indicated less than 10% positive tumor cells, 2 points indicated 10%–50% positive tumor cells, and 3 points indicated more than 50% positive tumor cells. In terms of intensity, no staining scored 0 points, weak staining scored 1 point, moderate staining scored 2 points, and strong staining scored 3 points. We added up the intensity score and proportion score and obtained the final score. The expression of specific gene and survival information from TCGA_CHOL were used for the Kaplan–Meier overall survival analysis with the medium levels of these genes as cut-off points. The gene expressions from TCGA_CHOL and GSE32879 [48] were used for correlation analysis. The gene expressions from TCGA_CHOL, GSE76297 [49], and GSE57555 [50] were used to compare the gene expression differences between tumor tissues and matched adjacent non-tumor tissues. The sources of these gene expression profiling datasets are listed in Table S6. 

### 4.15. Next-Generation Sequencing and Data Analysis

Total RNA was isolated from RBE cells infected with pLKO.1-shTESC or pLKO.1-Screamble control viruses and ligated to an adaptor for further amplification (Illumina^®^ TruSeqTM Stranded mRNA, San Diego, CA, USA) following assessment of RNA quality and quantity with a Bioanalyzer 2100 system (Agilent, Santa Clara, CA, USA) and fluorometry (Life Technologies, Qubit^®^ 2.0 Fluorometer, Waltham, MA, USA). All samples were sequenced on a NextSeq system (Illumina, San Diego, CA, USA). After sequencing, the read files (fastq) were mapped to the Hg38 reference by HISAT2 [51,52] and reads were counted using featureCounts [53]. Significantly enriched gene sets were analyzed using gene set enrichment analysis (GSEA, [54]), and the gene network was analyzed using ConsensusPathDB [55]. 

### 4.16. Statistical Analysis

Overall survival curves were plotted using the Kaplan–Meier method and compared using the log-rank test. The association between TESC expression and tumor size was evaluated using Pearson’s Chi-square test. All statistical analyses were performed using SPSS software, version 19 (SPSS, Inc., Chicago, IL, USA). A value of *p* <  0.05 was considered statistically significant. The OS-associated gene list was obtained by analyzing all genes and corresponding clinicopathological parameters from TCGA-CHOL database through Python lifeline package (v0.24.1) and listing genes that were significantly associated with poor survival.

## 5. Conclusions

In this study, we observed that high expression of TESC is associated with poor prognosis of patients with cholangiocarcinoma. We also demonstrated that TESC promotes cholangiocarcinoma tumor cell growth in vitro and in vivo. Elevated cellular levels of *TESC* are correlated with larger tumor size in cholangiocarcinoma. In terms of the regulatory mechanism of TESC, we found that TGF-α/EGFR signaling induces TESC via STAT3 binding directly on the *TESC* promoter and activating *TESC* transcription. Upon TGF-α induction, TESC is upregulated and promotes tumor growth through FOXM1-mediated G2/M transition. Our findings provide novel insight into TESC-mediated oncogenesis and reveal that TESC is a potential biomarker and therapeutic target for cholangiocarcinoma.

## Supplementary Materials

The following are available online at https://www.mdpi.com/2072-6694/12/5/1105/s1, Figure S1: Identification of genes that contribute to tumorigenesis in cholangiocarcinoma, Figure S2: TESC expression profile in the TCGA database, Figure S3: The expression of *CHP1* and *CHP2* in ICC, Figure S4: TESC is expressed in caerulein-treated rats, Figure S5: TESC regulates tumor proliferation, Figure S6: Knockdown of TESC inhibits the ability of migration, Figure S7: FOXM1 regulates tumor growth, Figure S8: TESC regulates the G2/M transition through FOXM1, Figure S9: Inhibition of EGFR-STAT3 signaling blocked TGF-α-induced TESC expression, Figure S10: Hematoxylin and eosin (H&E) and immunohistochemical staining of TESC and FOXM1, Figure S11: Doxycycline-inducible knockdown of TESC impairs colony formation in vitro, Figure S12: Immunoblotting files with densitometry data, Table S1: Reagents used in this study, Table S2: Primers used for qPCR, Table S3: Primers used for ChIP, Table S4: Antibodies used for immunoblotting, Table S5: shRNA clones used in this study, Table S6: Gene expression profiling data used in this study.
