# Supplementary material for: TESC Promotes TGF-α/EGFR-FOXM1-Mediated Tumor Progression in Cholangiocarcinoma
Source: Cancers (Basel). 2020 Apr 29;12(5):1105. doi: 10.3390/cancers12051105 (PMC7281536; doi:10.3390/cancers12051105)
Supplement: Supplementary file 1 [file cancers-12-01105-s001.pdf]

Supplementary Materials

TESC Promotes TGF-α/EGFR-FOXM1-Mediated Tumor Progression in Cholangiocarcinoma

Cheng-Han Hsieh, Cheng-Ying Chu, Sey-En Lin, Yu-Chen S.H. Yang, Hung-Shu Chang and Yun Yen

a.

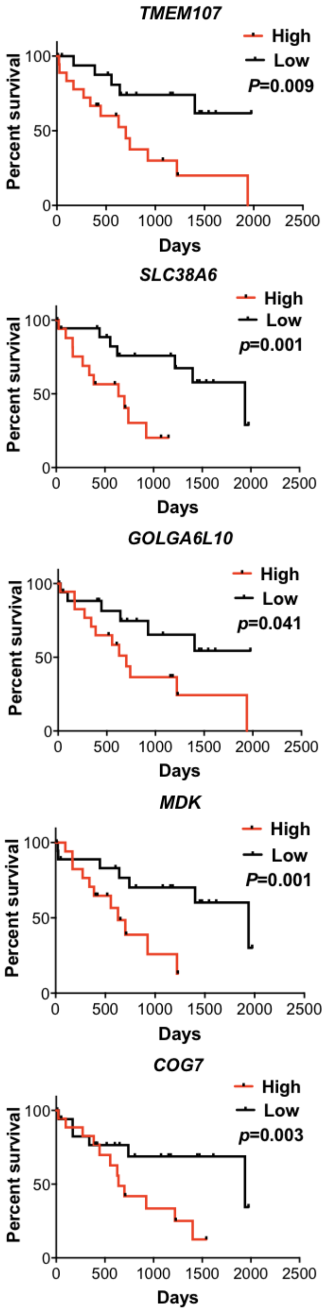

b.

n=36		pT stages	
p=0.371		T1	T2-T4
TMEM107 expression	Low	11 (57.9%)	7 (41.2%)
	High	8 (42.1%)	10 (58.8%)

n=36		pT stages	
p=0.738		T1	T2-T4
SLC38A6 expression	Low	10 (57.9%)	8 (41.2%)
	High	9 (42.1%)	9 (58.8%)

n=36		pT stages	
p=0.738		T1	T2-T4
GOLGA6L10 expression	Low	10 (57.9%)	8 (41.2%)
	High	9 (42.1%)	9 (58.8%)

n=36		pT stages	
p=0.738		T1	T2-T4
MDK expression	Low	10 (57.9%)	8 (41.2%)
	High	9 (42.1%)	9 (58.8%)

n=36		pT stages	
p=0.371		T1	T2-T4
COG7 expression	Low	11 (57.9%)	7 (41.2%)
	High	8 (42.1%)	10 (58.8%)

Figure S1. Identification of genes that contribute to tumorigenesis in cholangiocarcinoma (**a**) Kaplan–Meier analysis of candidate genes including *TMEM107*, *SLC38A6*, *GOLGA6L10*, *MDK*, and *COG7* with overall survival in 36 cholangiocarcinoma patients from TCGA database. (**b**) Chi-square analysis of the association between candidate genes and pT stages in cholangiocarcinoma from the TCGA database.

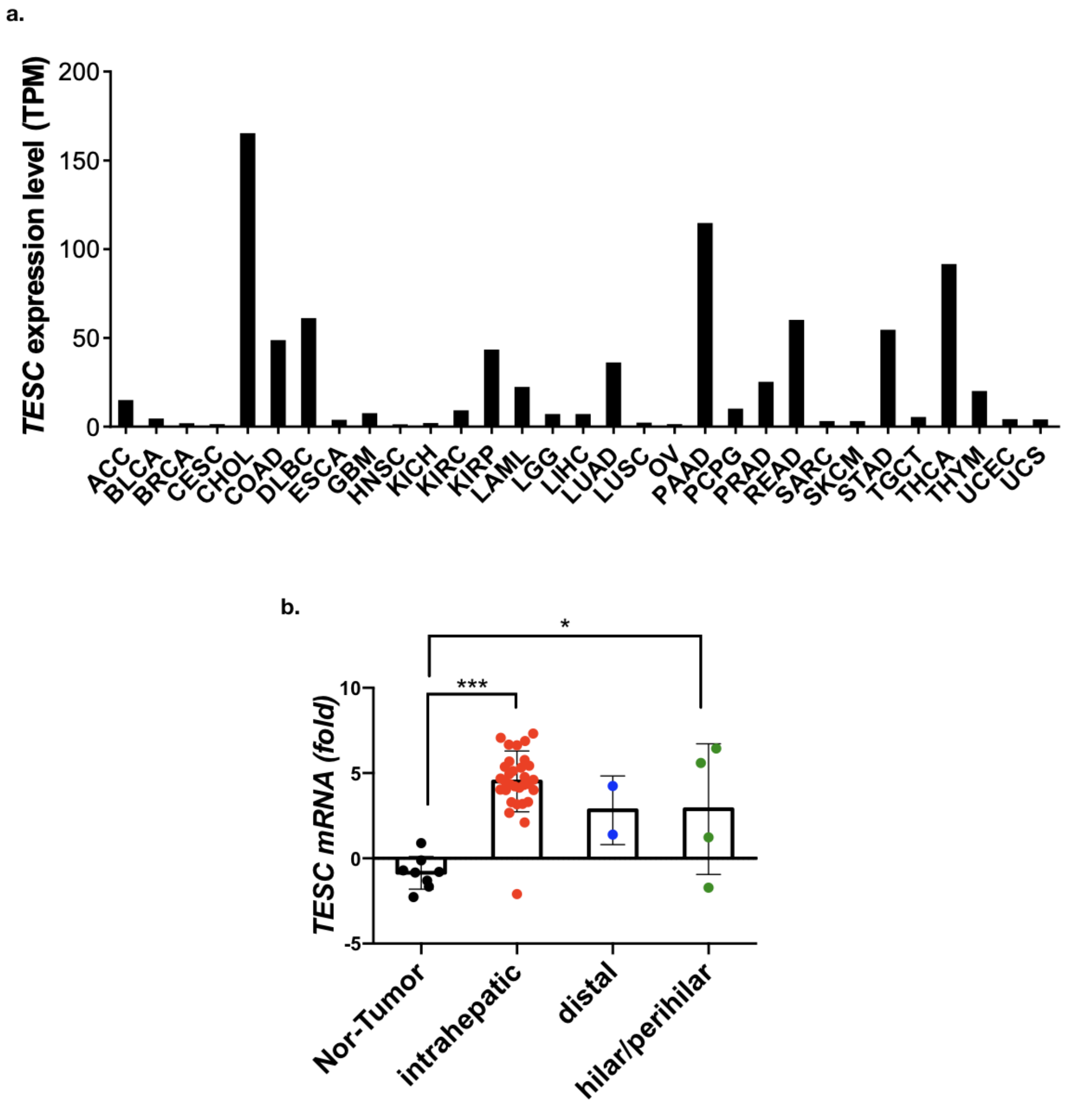

Figure S2. *TESC* expression profile in TCGA database. (**a**) The gene expression profile of *TESC* across all tumor samples, which were derived from TCGA databases. Data were downloaded from GEPIA database (http://gepia.cancer-pku.cn/index.html). ACC, adrenocortical carcinoma; BLCA, bladder urothelial carcinoma; BRCA, breast invasive carcinoma; CESC, cervical squamous cell carcinoma and endocervical adenocarcinoma; CHOL, cholangiocarcinoma; COAD, colon adenocarcinoma; DLBC, lymphoid neoplasm diffuse large B-cell lymphoma; ESCA, esophageal carcinoma; GBM, glioblastoma multiforme; HNSC, head and neck squamous cell carcinoma; KICH, kidney chromophobe; KIRC, kidney renal clear cell carcinoma; KIRP, kidney renal papillary cell carcinoma; AML, acute myeloid leukemia; LGG, brain low-grade glioma; LIHC, liver hepatocellular carcinoma; LUAD, lung adenocarcinoma; LUSC, lung squamous cell carcinoma; MESO, mesothelioma; OV, ovarian serous cystadenocarcinoma; PAAD, pancreatic adenocarcinoma; PCPG, pheochromocytoma and paraganglioma; PRAD, prostate adenocarcinoma; READ, rectum adenocarcinoma; SARC, sarcoma; SKCM, skin cutaneous melanoma; STAD, stomach adenocarcinoma; TGCT, testicular germ cell tumors; THCA, thyroid carcinoma; THYM, thymoma; UCEC, uterine corpus endometrial carcinoma; UCS, uterine carcinosarcoma; and UVM, uveal melanoma. (**b**) The level of *TESC* in different pathological subtypes of cholangiocarcinoma, which were derived from TCGA database. * $p < 0.05$, *** $p < 0.001$.

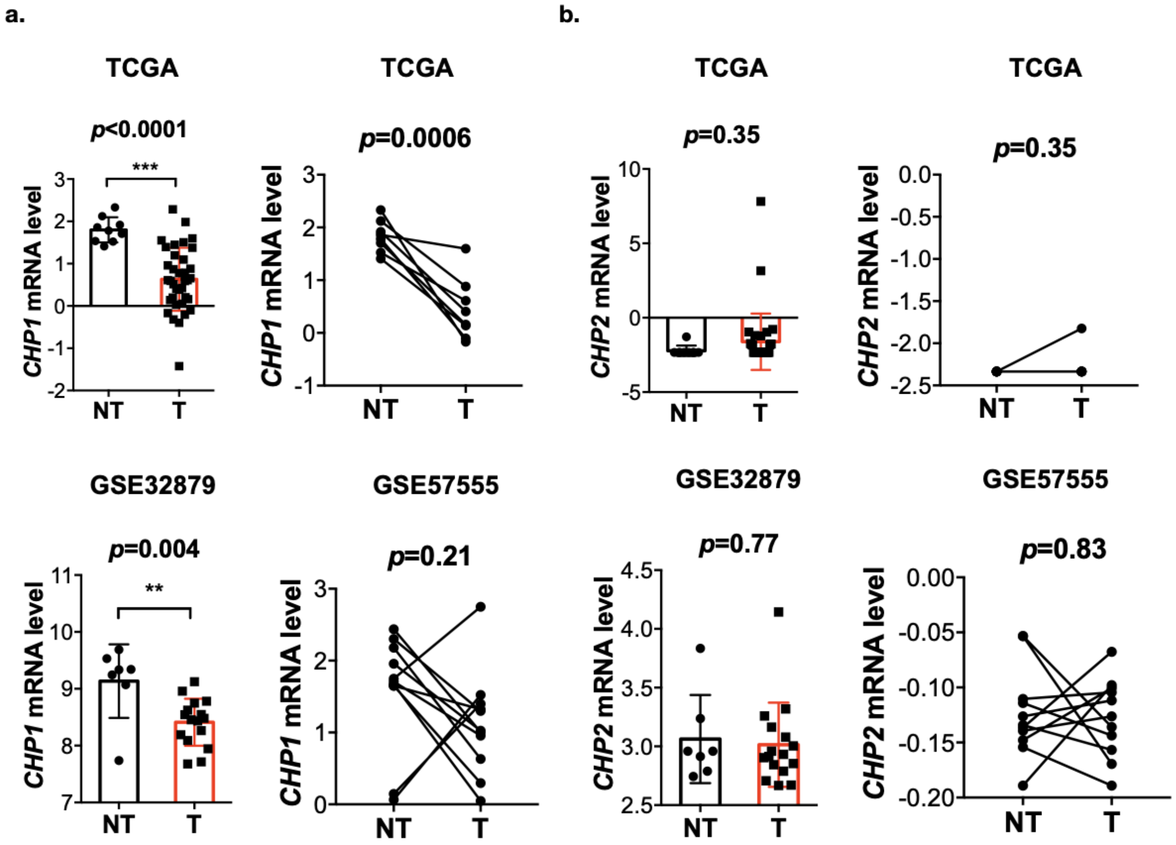

Figure S3. The expression of *CHP1* and *CHP2* in ICC. (**a**) Scatter plots (left) and line plots (right) of differential expression of *CHP1* in tumor (T) tissues versus non-tumor (NT) tissues from the TCGA (top) and GEO (bottom) databases. ** $p < 0.01$, *** $p < 0.001$. (**b**) Scatter plots (left) and line plots (right) of differential expression of *CHP2* in tumor (T) tissues versus non-tumor (NT) tissues from the TCGA (top) and GEO (bottom) databases.

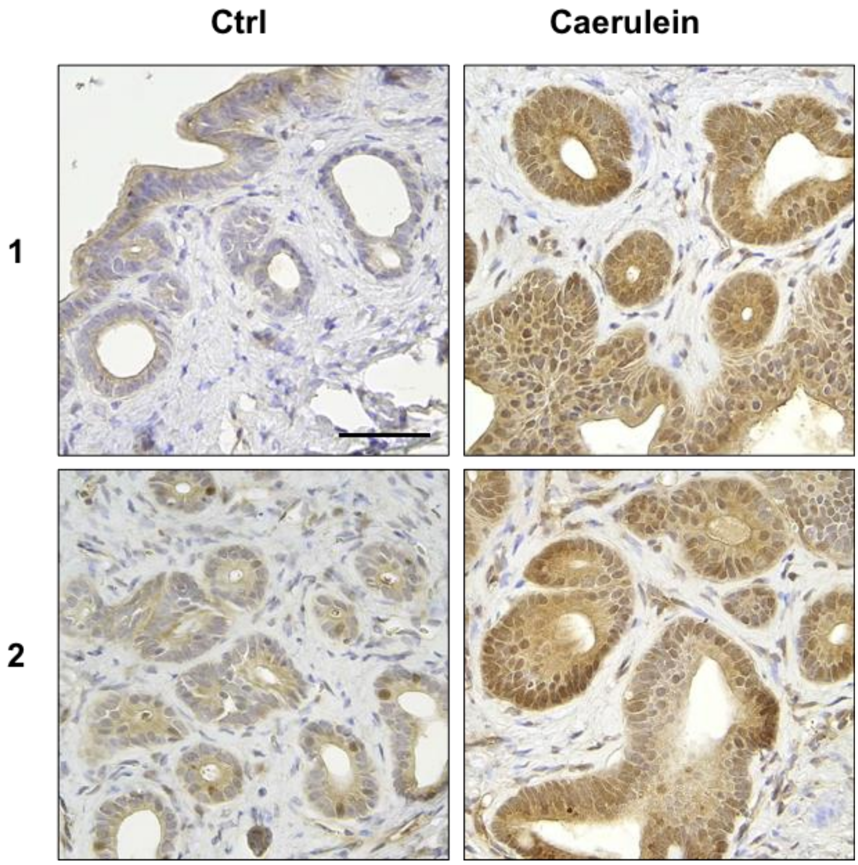

Figure S4. TESC is expressed in caerulein-treated rats. Representative results of immunohistochemical staining for TESC in mice treated with or without caerulein. Black scale bars, 50 μm.

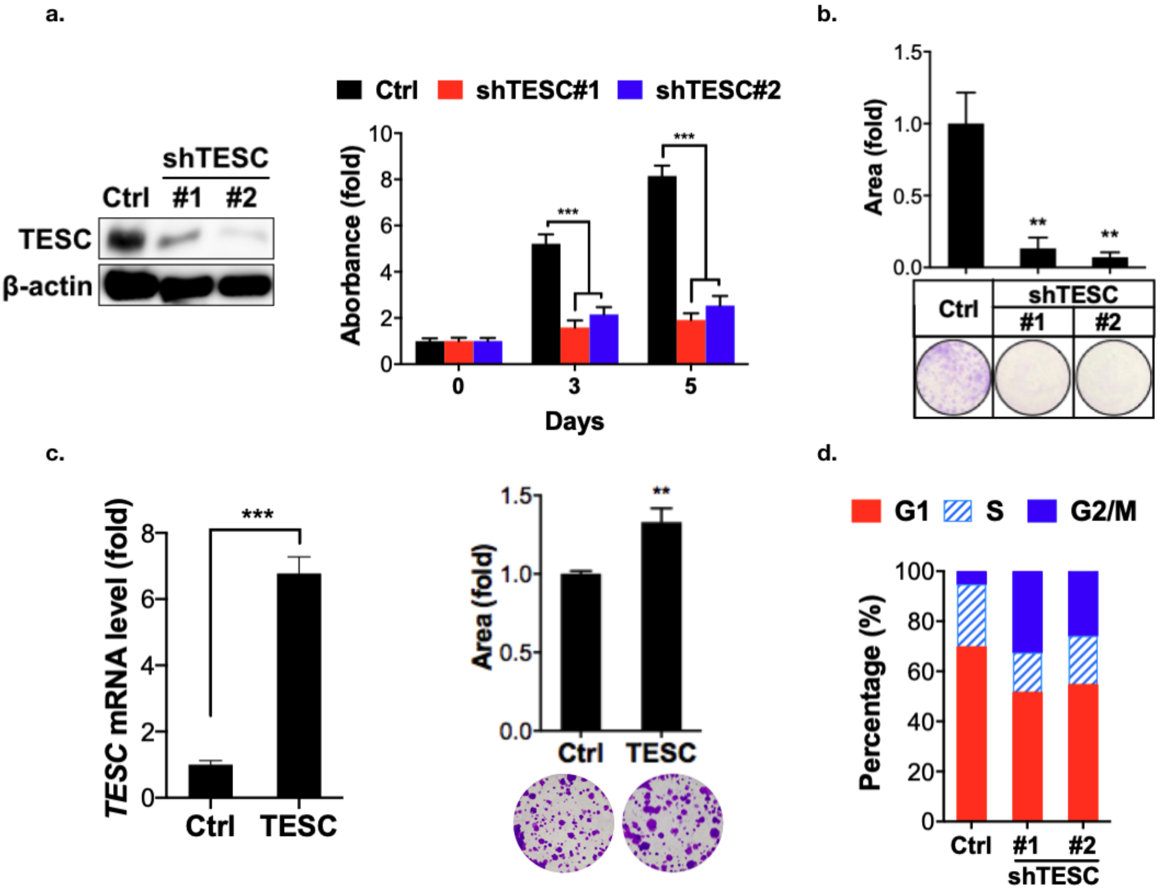

Figure S5. TESC regulates tumor proliferation. (**a**) MTT analysis of proliferation of HUCCT1 cells infected with lentiviral vectors encoding shTESC or scrambled control (Ctrl). The level of TESC was assessed through immunoblotting (left). #1 and #2 indicate distinct shRNAs that target different regions within TESC. Results are representative of three independent experiments and expressed as the mean ± SD; *** $p < 0.001$. (**b**) Clonogenic assays of HUCCT1 cells infected with lentiviral vectors encoding shTESC or scrambled control for 14 days. Top: Colonies were stained with crystal violet and quantified. Bottom: Representative plates were photographed. ** $p < 0.01$. (**c**) Clonogenic assays of HUCCT1 cells infected with lentiviral vectors encoding TESC or vector control for 14 days. Top: Colonies were stained with crystal violet and quantified. Bottom: Representative plates were photographed. ** $p < 0.01$, *** $p < 0.001$ (**d**) Flow cytometric analysis of the loss of the TESC effect on the cell cycle in HUCCT1 cells infected with lentiviral vectors encoding shTESC or scrambled control for 5 days.

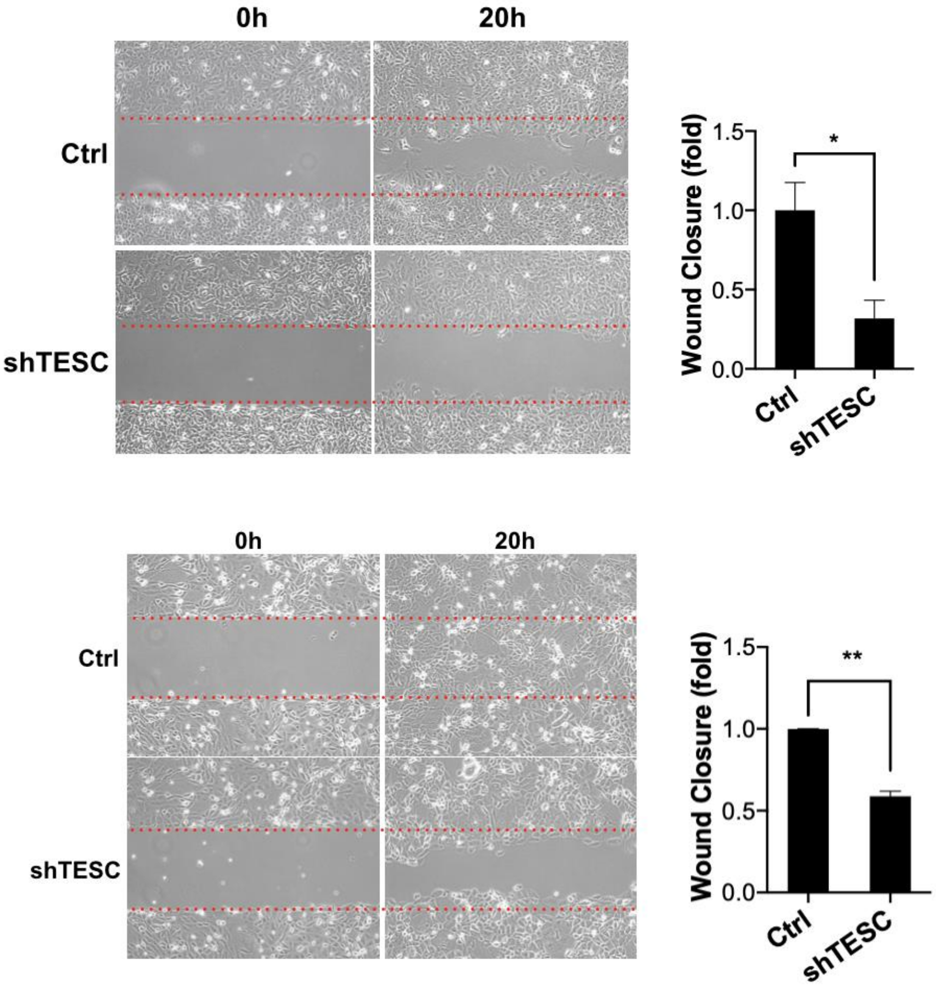

Figure S6. Knockdown of TESC inhibits the ability of migration. The migration ability of RBE (upper) and HUCCT1 (bottom) cells infected with lentiviral vectors encoding shTESC, or scrambled control (ctrl) were assessed by wound-healing assay. Images were obtained at 0 and 20 h (left) and quantified (right). * $p < 0.05$, ** $p < 0.01$.

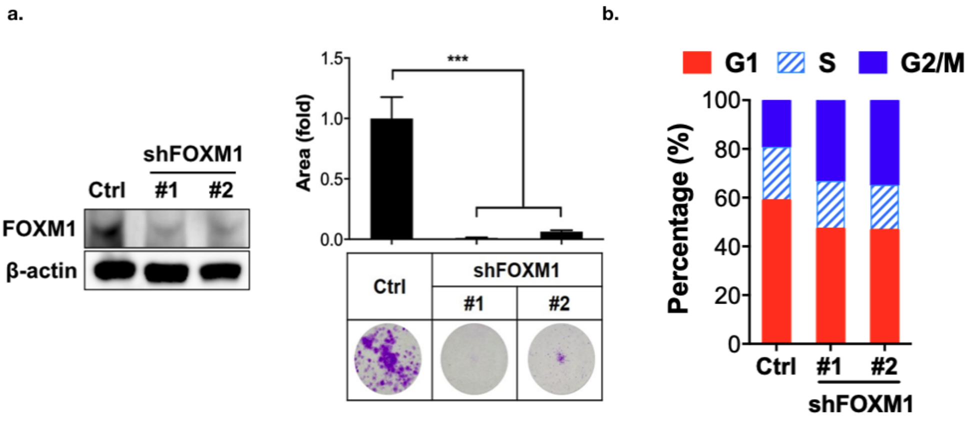

Figure S7. FOXM1 regulates tumor growth. (**b**) Clonogenic assays of RBE cells infected with lentiviral vectors encoding shFOXM1 or scrambled control (Ctrl) for 14 days. Top: Colonies were stained with crystal violet and quantified. Bottom: Representative plates were photographed. The level of FOXM1 was assessed through immunoblotting (left). *** $p < 0.001$. (**b**) Flow cytometric analysis of the loss of the FOXM1 effect on the cell cycle in RBE cells infected with lentiviral vectors encoding shFOXM1 or scrambled control for 5 days.

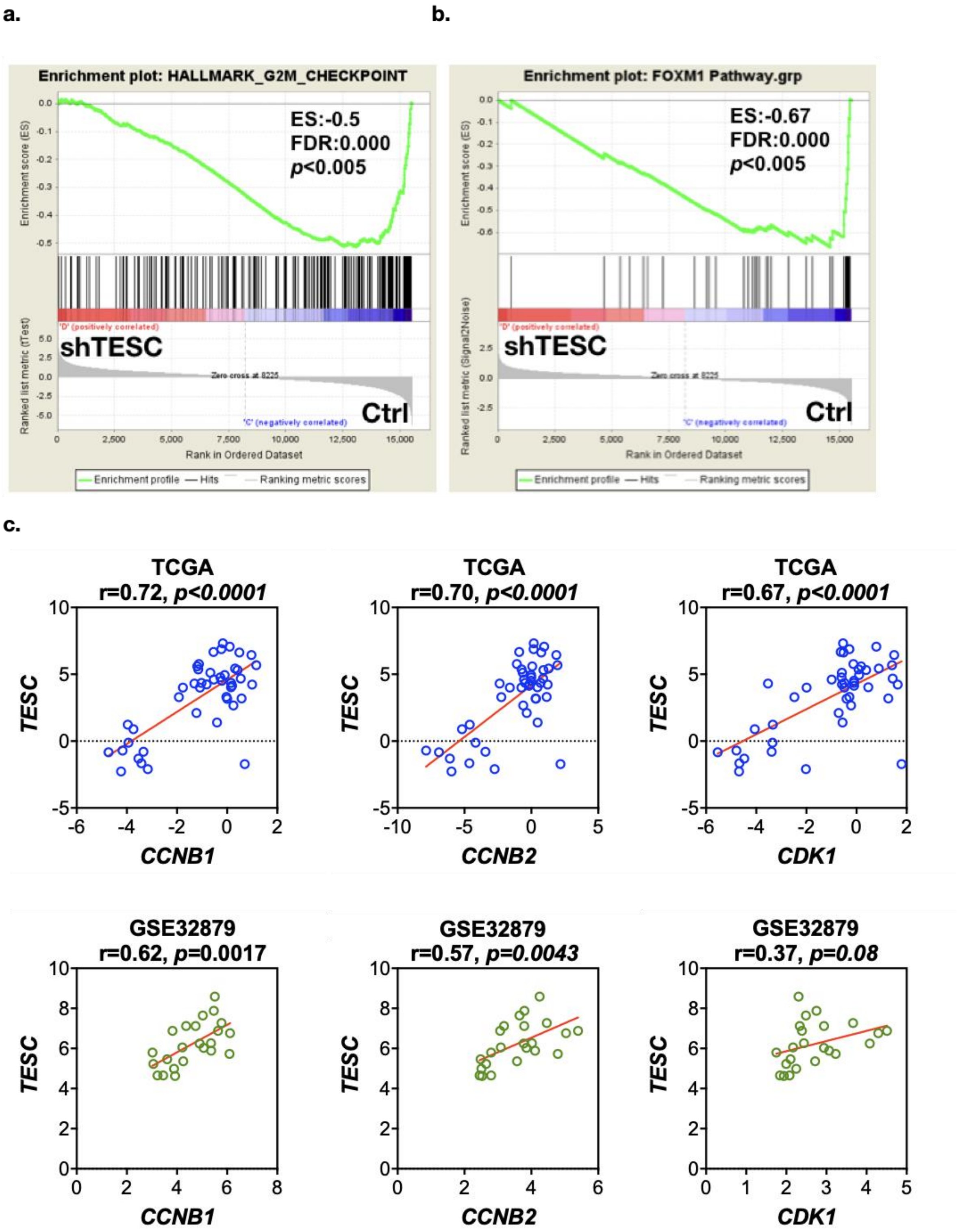

Figure S8. TESC regulates the G2/M transition through FOXM1. (**a**) GSEA analyses showing enrichment of human G2M checkpoint-related genes in reference signatures comparing RBE cells infected with lentiviral vectors encoding shTESC or vector control (Ctrl). (**b**) GSEA analyses showing enrichment of human FOXM1 target genes in reference signatures comparing RBE cells infected with lentiviral vectors encoding TESC or vector control (Ctrl). (**c**) Dot-plot analyses for co-expression between *TESC* and *CCNB1*, *CCNB2*, or *CDK1* in cholangiocarcinoma from the TCGA (top) and GEO (GSE32879, bottom) databases.

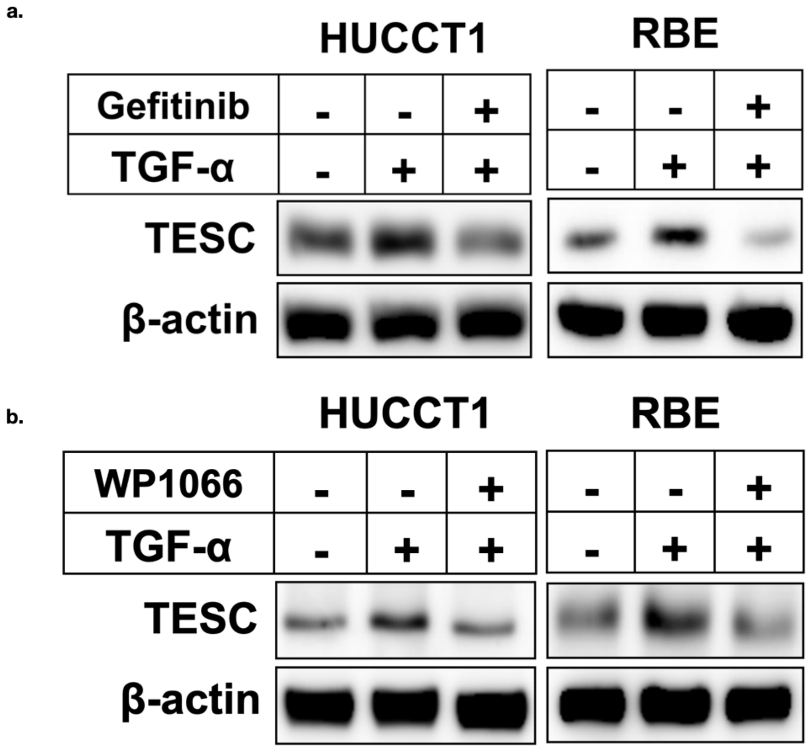

Figure S9. Inhibition of EGFR-STAT3 signaling blocked TGF-α-induced TESC expression. (**a**) immunoblotting analysis of TESC expression in HUCCT1 (left) and RBE (right) cells treated with or without TGF-α (50 ng/mL) in the present or absence of gefitinib (5 μM) for 8 h. β-actin was assessed as an internal control. (**b**) Immunoblotting analysis of TESC expression in HUCCT1 (left) and RBE (right) cells treated with or without TGF-α (50 ng/mL) in the presence or absence of WP1066 (5 μM) for 8 h. β-actin was assessed as an internal control.

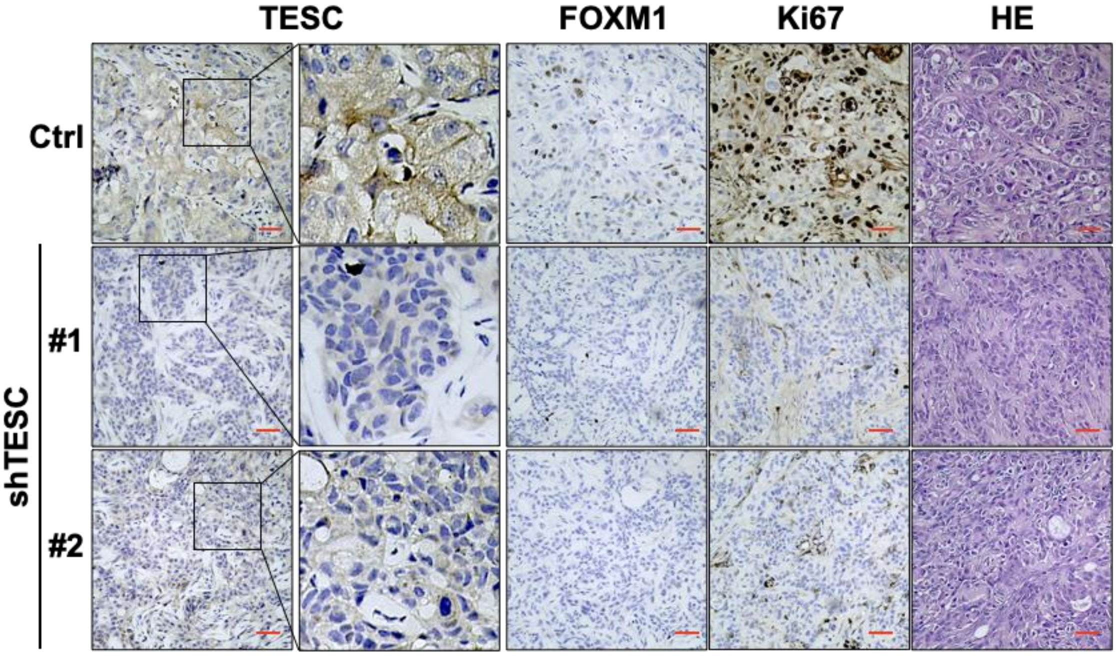

Figure S10. Hematoxylin and eosin (H&E) and immunohistochemical staining of TESC and FOXM1. Excised tumors from mice in Figure 7b were further subjected to H&E staining combined with immunohistochemistry analysis. Red scale bar, 50 μm.

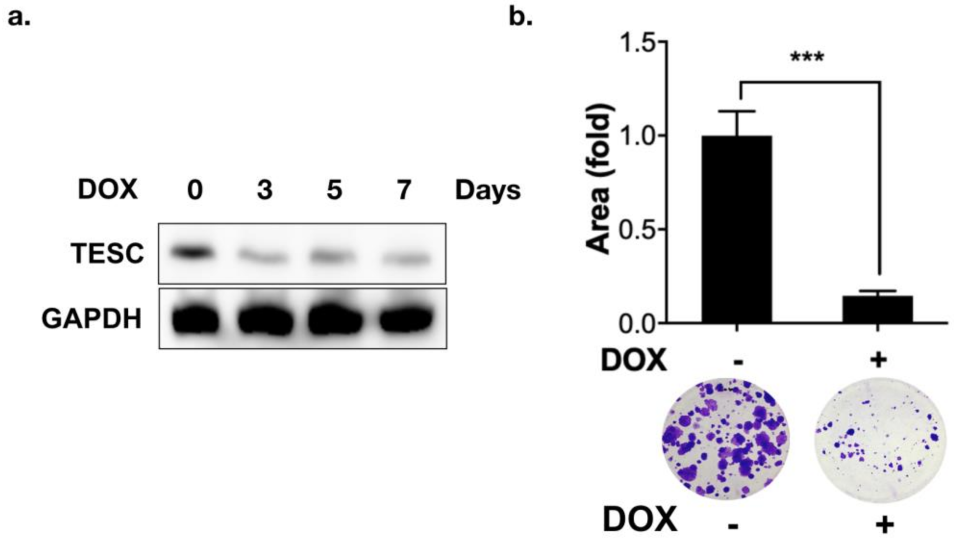

Figure S11. Doxycycline-inducible knockdown of TESC impairs colony formation *in vitro*. (**a**) Immunoblotting for TESC in HUCCT1-tet_on-shTESC cells in the present or absence of doxycycline (DOX) for the indicated times. (**b**) Clonogenic assays of HUCCT1-tet_on-shTESC HUCCT1 cells treated with or without DOX for 14 days. Upper: Colonies were stained with crystal violet and quantified. Bottom: Representative plates were photographed. *** $p < 0.001$.

Figure 3b

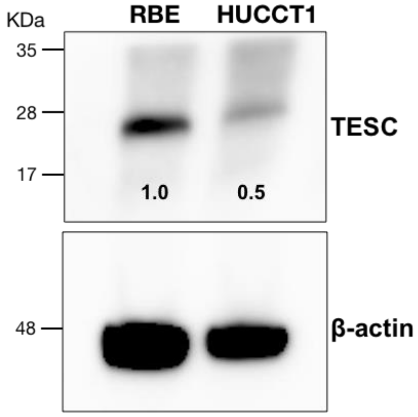

Figure 3c

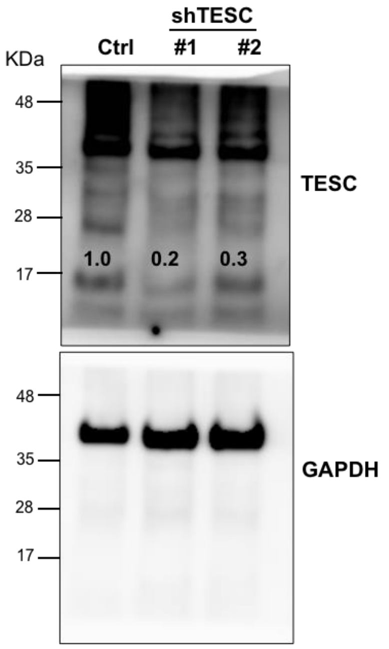

TESC antibody (11125-1-AP), Proteintech
Expected band at 25~30KDa

Figure 3f

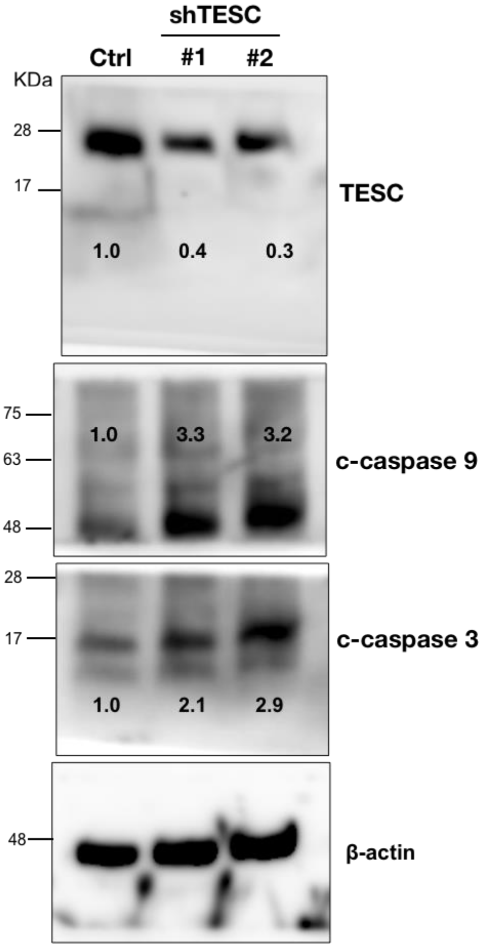

Figure S12. *Cont.*

Figure 4d

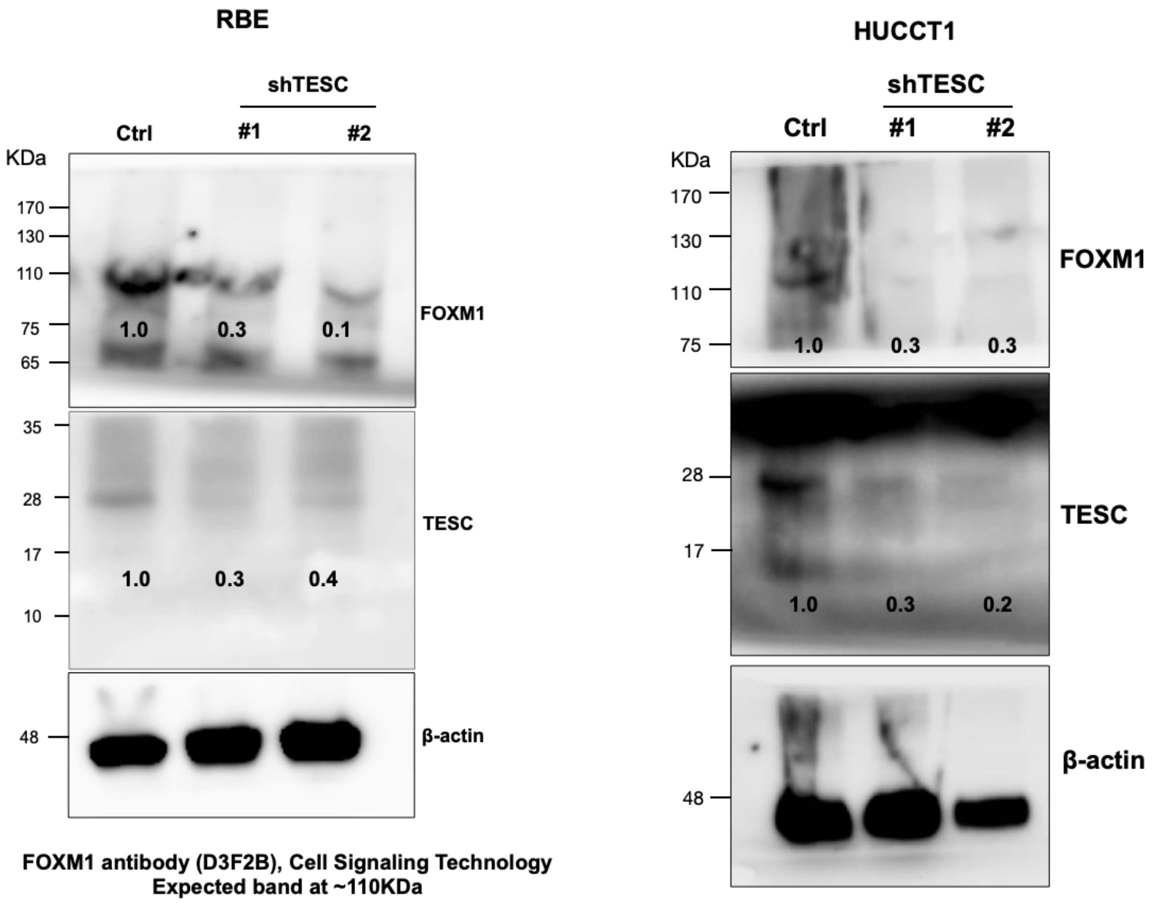

Figure 4e

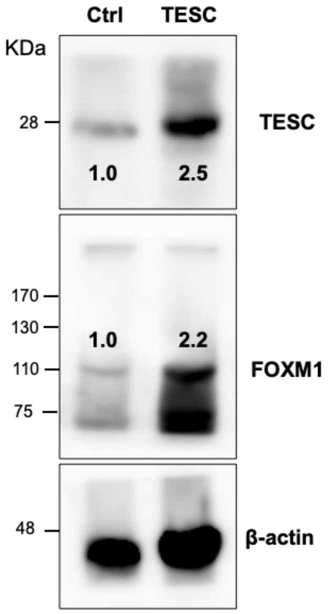

Figure S12. *Cont.*

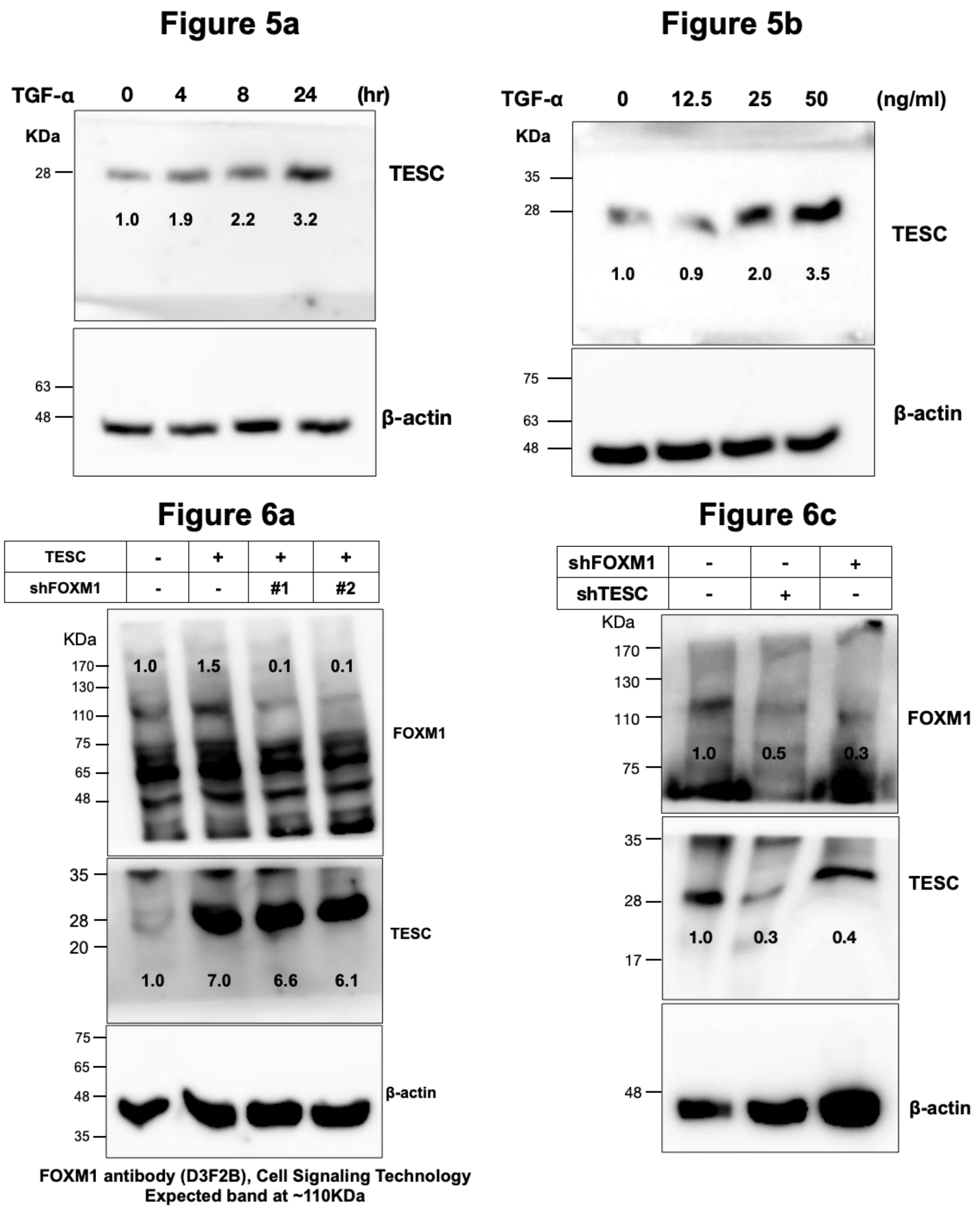

Figure S12. *Cont.*

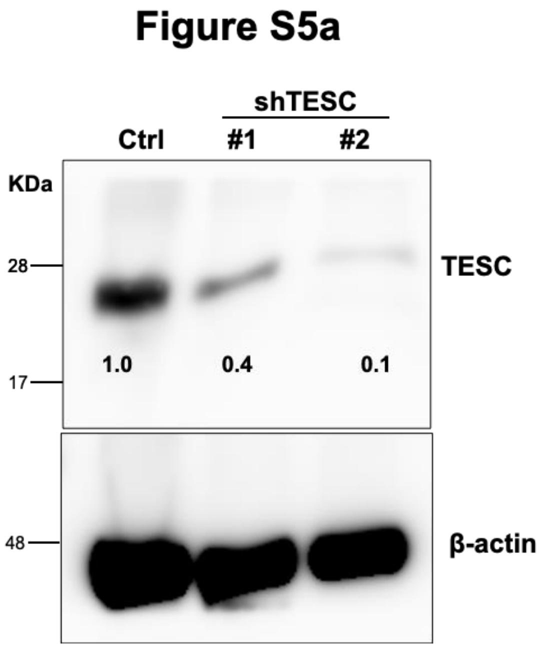

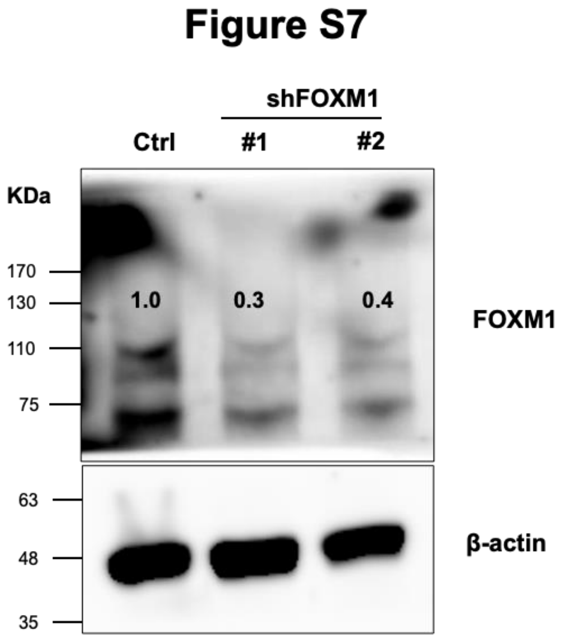

Figure S12. *Cont.*

Figure S9

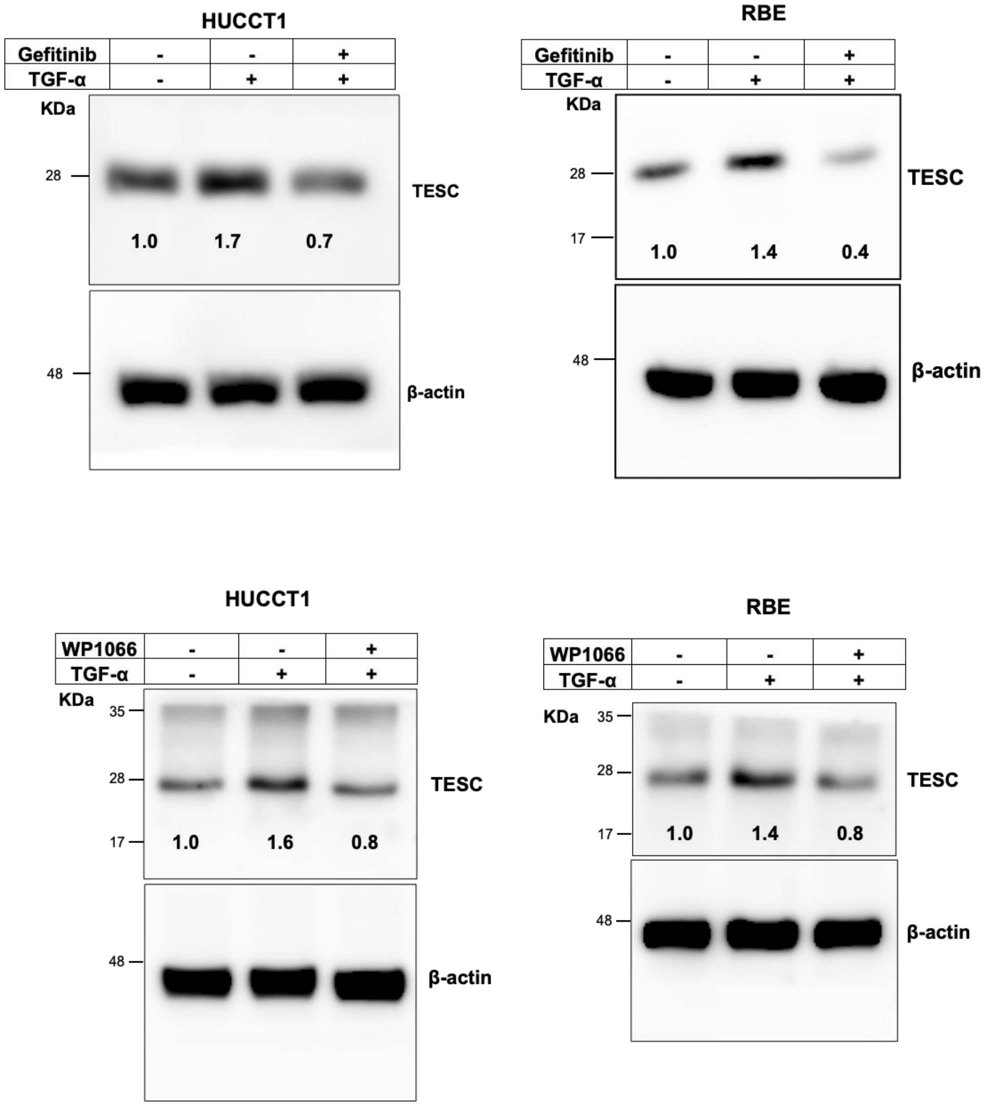

Figure S11

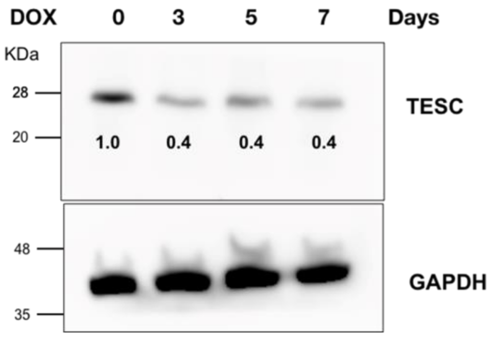

Figure S12. Immunoblotting files with densitometry data.

Table S1. Reagents used in this study.

Reagent	Manufacturer
Recombinant human TGF-α	Sino Biological (Beijing, China)
Gefitinib	LC Laboratories (Woburn, MA, USA)
WP1066	Cayman Chemical (Ann Arbor, MI, USA)
Doxycycline hyclate	GOLD BIO (St. Louis, MO, USA)

Table S2. Primers used for qPCR.

Q-PCR primer	Sequence
18S	Forward sequence: 5′-GACCAGAGCGAAAGCAT-3′<br>Reverse sequence: 5′-TCGGAACTACGACGGTATC-3′
TESC	Forward sequence: 5′-CTGAGTGGAGATCAGCCTACCA-3′<br>Reverse sequence: 5′-CTGTTGTCGAAGAAGGCACGAAC-3′
FOXM1	Forward sequence: 5′-TCTGCCAATGGCAAGGTCTCCT-3′<br>Reverse sequence: 5′-CTGGATTCGGTCGTTTCTGCTG-3′
EGFR	Forward sequence: 5′-AACACCCTGGTCTGGAAGTACG-3′<br>Reverse sequence: 5′-TCGTTGGACAGCCTTCAAGACC-3′
STAT3	Forward sequence: 5′-CTTTGAGACCGAGGTGTATCACC-3′<br>Reverse sequence: 5′-GGTCAGCATGTTGTACCACAGG-3′

Table S3. Primer used for ChIP.

ChIP primer	Sequence
TESC promoter	Forward sequence: 5′-GTGTGTTCACCTCAGAAG-3′<br>Reverse sequence: 5′-GAGATAGTCAAGGAAGTCAG-3′

Table S4. Antibodies used for Weston blotting.

Antibody	Catalog Number	Manufacturer
anti-TESC	11125-1-AP	Proteintech (Rosemont, IL, USA)
anti-β-actin	GTX109639	GeneTex (Irvin, CA, USA)
anti-GAPDH	GTX100118	GeneTex (Irvin, CA, USA)
anti-FOXM1	D3F2B	Cell Signaling Technology (Danvers, MA, USA)

Table S5. shRNA clones used in this study.

TRC clone	Clone ID	Target sequence
pLKO.1-TESC #1	TRCN0000299837	CCTGGCTGATGAGATCAATTT
pLKO.1-TESC #2	TRCN0000299838	AGTGGAGATCAGCCTACCATT
pLKO.1-FOXM1 #1	TRCN0000273982	GCCAATCGTTCTCTGACAGAA
pLKO.1-FOXM1 #2	TRCN0000273981	AGGACCACTTTCCCTACTTTA
pLKO.1-STAT3	TRCN0000020842	GCACAATCTACGAAGAATCAA
pLKO.1-EGFR	TRCN0000039634	GCTGGATGATAGACGCAGATA

Table S6. Gene expression profiling data used in this study.

Cohort	Source	References
TCGA_CHOL	TCGA	https://portal.gdc.cancer.gov/projects/TCGA-CHOL
GSE76297	GEO	https://www.ncbi.nlm.nih.gov/geo/query/acc.cgi?acc=GSE76297
GSE32879	GEO	https://www.ncbi.nlm.nih.gov/geo/query/acc.cgi?acc=GSE32879
GSE57555	GEO	https://www.ncbi.nlm.nih.gov/geo/query/acc.cgi?acc=GSE57555

© 2020 by the authors. Licensee MDPI, Basel, Switzerland. This article is an open access article distributed under the terms and conditions of the Creative Commons Attribution (CC BY) license (http://creativecommons.org/licenses/by/4.0/).
